# Clinical grade expansion protocol for the manufacture of thymus-derived Treg cells for clinical application

**DOI:** 10.1186/s12967-025-06561-9

**Published:** 2025-06-03

**Authors:** Giorgia Fanelli, Philippa Marks, Apoorva Aiyengar, Marco Romano, Sakina Gooljar, Sandeep Kumar, Michael Burch, Giovanna Lombardi

**Affiliations:** 1 Peter Gorer Department of Immunobiology, School of Immunology and Microbial Sciences, King’s College, London, UK; 2 Department of Cardiology, Great Ormond Street Hospital NHS Foundation Trust, London, UK; 3 Research Department of Children’s Cardiovascular Disease, Institute of Cardiovascular Science, University College London, London, UK; 4 Advanced Therapy Manufacturing (GMP) Unit Guy’s, St Thomas’ NHS Foundation Trust and King’s College London Clinical Research Facility, London, UK

**Keywords:** Thy-Tregs, Immuno-therapy, GMP, Clinical trial

## Abstract

**Background:**

Adoptive transfer of regulatory T cells (Tregs) has provided promising results in treating autoimmune disorders, transplant rejection and graft versus-host disease in early clinical trials. However, major challenges remain for developing a standardized and robust good manufacturing practice (GMP)-compliant cell product which is severely hampered by low frequency of Tregs in circulation and laborious ex vivo expansion.

**Methods:**

Paediatric thymuses routinely obtained during heart surgery have been shown by us and others to be a valuable source of large numbers of pure Tregs (Thy-Tregs). Here we show results from our process development approach including systematic laboratory-scale testing of activation reagents, restimulation timing, and cryopreservation to translate our expansion protocol of Thy-Tregs into a clinical grade cell product.

**Results:**

Thy-Tregs obtained through CD8^+^ cell depletion and subsequent CD25^+^ enrichment were expanded with αCD3/αCD28 beads in the presence of Rapamycin and IL-2 for 10–23 days using G-Rex bioreactors. We successfully embedded bead removal and final formulation of a cryopreserved cell product ready to be used at bedside transfusion.

**Conclusion:**

This process has proved the capability of efficiently producing high number of functional Thy-Tregs, which will be administered as cell therapy in children undergoing heart transplantation (ATT-Heart, ISRCTN15374803), and enhancing the potential of using expanded Thy-Tregs for broad-ranging therapeutic applications.

**Supplementary Information:**

The online version contains supplementary material available at 10.1186/s12967-025-06561-9.

## Introduction

Over the past years, the success of Treg transfer in preclinical animal models of GvHD, solid organ transplantation and autoimmune diseases has laid the foundations for several early-stage clinical trials aiming to investigate the safety and biological impact of adaptive Treg therapy [1–3]. Collectively, phase I clinical trials using Tregs have shown promising results, proving the feasibility and safety of infusing large numbers of ex vivo expanded Tregs [4–11]. Though extensive work has been done to develop the best approach to manufacturing Treg cell products, there are still several open questions regarding the source, isolation method and culture conditions to generate large numbers of stable and functional Tregs, while minimizing manipulation and manufacturing cost [12].

We and others have recently shown the possibility to isolate Tregs from discarded paediatric thymuses for clinical applications [13–15]. Indeed, CD4^+^CD25^+^ CD127^low^ Tregs isolated from thymus tissue (Thy-Tregs) are predominantly a naïve and homogenous population compared to the peripheral counterpart, and they maintain a stable phenotype under inflammatory conditions [13, 14, 16]. Such properties have made Thy-Tregs a feasible cell therapy for a phase I clinical trial currently ongoing in Spain (NCT04924491) where autologous Thy-Tregs are administered to infants undergoing heart transplantation [17] and they are currently under investigation as an allogeneic “off-the shelf” therapy to prevent graft versus disease (GVHD) following hematopoietic stem cell transplant (HSCT).

Despite the possibility of manufacturing highly pure Tregs from thymus tissue, the capability of expanding large number of Thy-Tregs for adoptive transfer is still a hurdle to overcome. While the presence of IL-2 remains a *conditio sine qua non* for proliferation and maintenance of Treg phenotype [14, 15, 18, 19], current Thy-Treg expansion protocols published by us and others [14, 15, 20] differ by multiple parameters including number of restimulations required and restimulation time resulting in highly variable fold expansion rates. Another complexity in Thy-Treg manufacturing is the possibility to cryopreserve expanded cells while maintaining stable FOXP3 expression and suppressive ability [20–22].

In the present study, we show data supporting the technology transfer of the Thy-Treg expansion protocol for manufacturing high cellular doses for a phase I clinical trial (ATT-Heart ISRCTN15374803) aiming to treat children (age range from 6 months to 16 years old) receiving heart transplant. We designed a manufacturing protocol which guarantees a highly pure and functional clinical Thy-Treg product with the possibility of harvesting the cells between 10 and 23 days upon expansion making such Treg product suitable for a broad cohort of children of different age.

In addition, we have validated a robust way to cryopreserve our expanded clinical grade Thy-Tregs which after freezing and thawing were still more than 95% viable and 80% FOXP3^+^. Such implementation will be beneficial not only for that cohort of patients undergoing later heart transplant following thymic retrieval at prior cardiac surgery such as implantation of mechanical support, but also for future cell therapy applications as potential “off-the shelf” allogeneic cell product.

## Materials and methods

### Tissue processing and Thy-Treg isolation

Thymus tissue samples were collected following cardiac surgery at “Great Ormond Street Hospital” at University College London (UCL) and stored up to 48 h at 4 °C before cell isolation (day 0 of culture protocol). Informed consent was obtained from all the donors prior to the tissue donation (ethical approval: 06-MI-13(B)). To generate Supplementary Fig. 1A, three thymus samples (donor 1, 10 and 11) were divided in two equal parts and the thymocyte suspension was obtained after either enzymatic digestion followed by the mechanical step using the gentleMACS Dissociator (Miltenyi Biotec) as previously described [14] or after using gentleMACS Dissociator only. All the donor samples listed in Table 1 were processed with the gentleMACS Dissociator (Miltenyi Biotec) only. Isolation of Thy-Tregs was performed as previously described in a two- step procedure [14].The same methodology was used to perform the engineering run under GMP conditions with clinical grade reagents (Supplementary Table 1) and the CliniMACS Plus separation system (Miltenyi Biotec). Briefly, after depletion of CD8 + cells using CliniMACS CD8 Reagent (Miltenyi Biotec, 275-01), the negative product was enriched for the CD25 positive fraction (CliniMACS CD25 Reagent, Miltenyi Biotec, 274-01).

### Thy-Treg expansion protocol

**Fig. 1:**
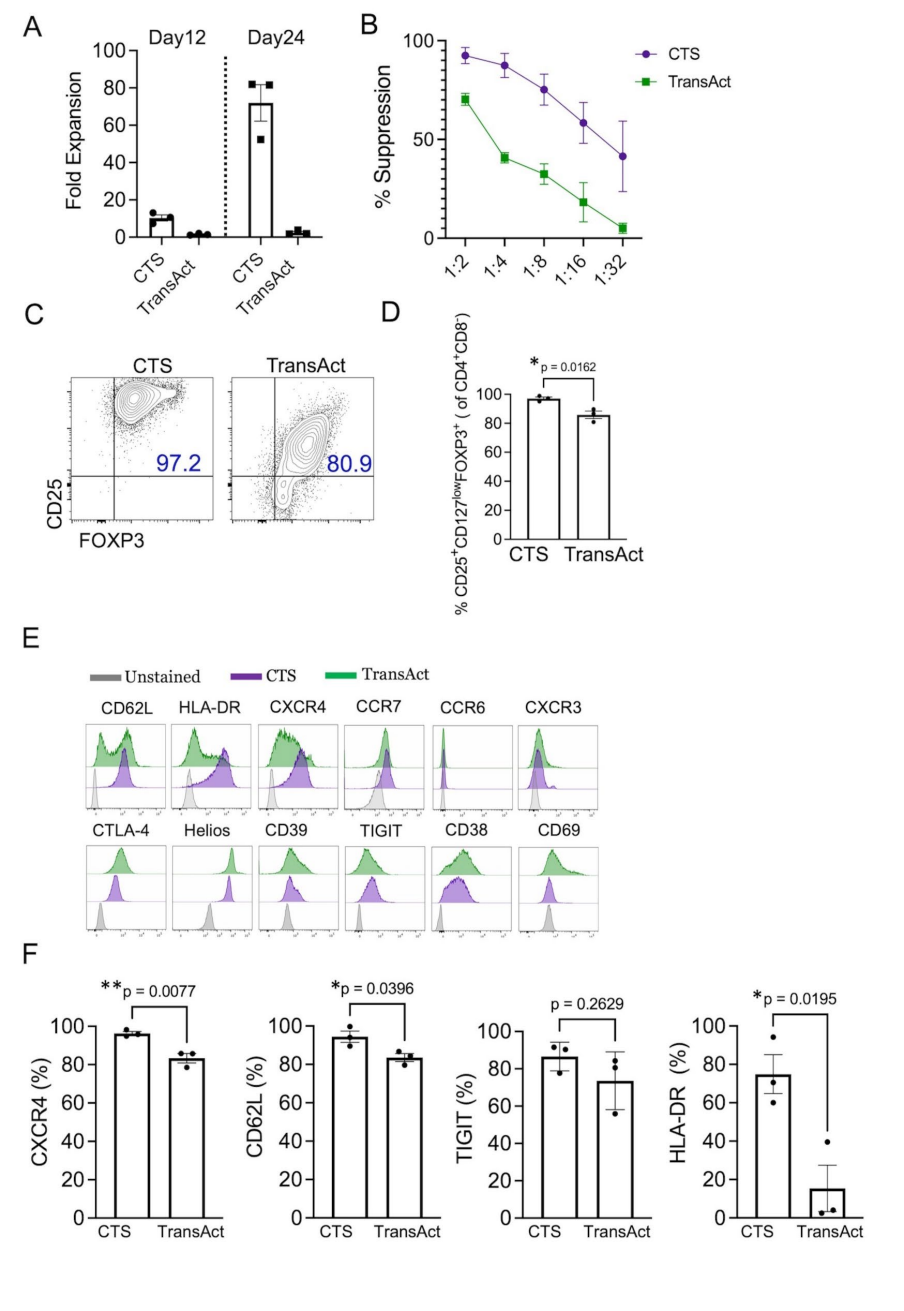
Activation reagents comparison to expand Thy-Tregs. **(A)** Fold expansion of Thy-Tregs restimulated at day 12 and expanded with CTS Treg Expander or TransAct for 24 days in flasks. **(B)** Suppressive ability of Thy-Tregs differently activated for 24 days and cocultured with CFSE-labelled effector CD25- T cells, HLA-A2 mismatched to Tregs, at the indicated ratios and stimulated with αCD3/αCD28 beads at 40:1 (cell/bead) ratio for 5 days. Suppression of CD25^−^ T cell proliferation was determined by division index. **(C)** Representative plots and cumulative data **(D)** showing CD25 and FOXP3 expression on Thy-Tregs stimulated as in (A). Bars represent the mean ± SEM. Data are pooled from 3 independent experiments (*n* = 3 different donors). *P < 0.05 was considered significant using two-tailed Unpaired t-test. **(E)** Representative histograms showing the expression of the indicated markers in the Thy-Tregs activated as in (A). **(F)** Quantification of CXCR4, CD62L, TIGIT and HLA-DR expression on Thy-Tregs shown in (E). Bars represent the mean ± SEM. Data are pooled from 3 independent experiments (*n* = 3 different donors). *P < 0.05 and ** *P* < 0.01 were considered significant using two-tailed Unpaired t-test

**Fig. 2:**
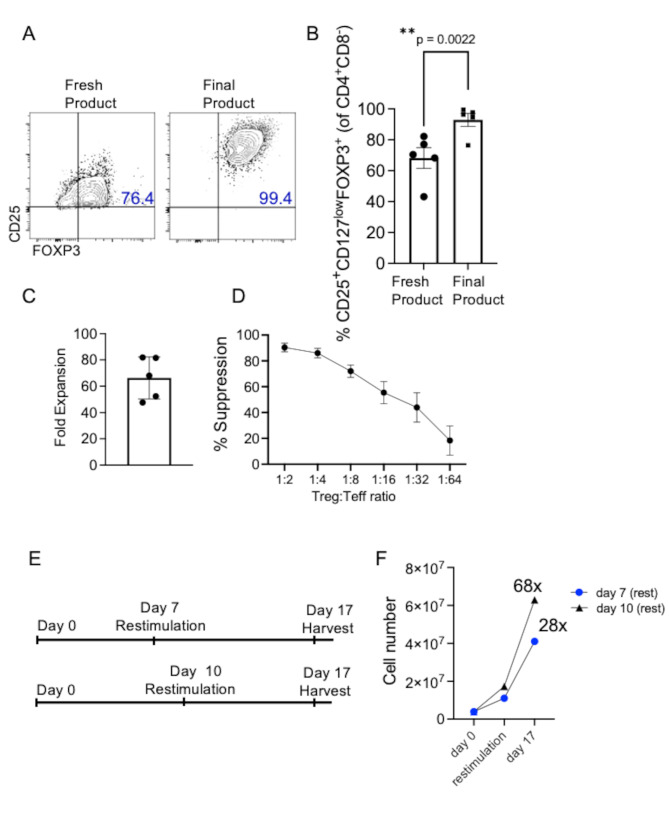
Validation of Thy-Teg expansion protocol on small-scale laboratory runs. **(A)** Representative plots and **(B)** quantification of CD4^+^CD25^+^CD127^low^FOXP3^+^ cells freshly isolated and following 24 days of expansion. Data are pooled from 5 independent experiments (*n* = 5 donors). Bars represent the mean ± SEM. **P < 0.01 was considered significant using paired *t* test. **(C)** Cumulative fold expansion from *n* = 5 donors (5 independent expansions) expanded in flasks for 24 days. Bar represents the mean ± SEM. **(D)** Suppressive ability of Thy-Tregs from the same donors in (C) cocultured with CFSE-labelled effector CD25^−^ T cells, HLA-A2 mismatched to Tregs, at the indicated ratios and stimulated with αCD3/αCD28 beads at 40:1 (cell/bead) ratio for 5 days. Suppression of CD25^−^ T proliferation was determined by division index (*n* = 5, from 5 independent experiments). **(E)** Schematic representation of the restimulation time comparison tested on Thy-Tregs cultured in a Grex-10 for 17 days and restimulated either at day 7 or at day 10. **(F)** Final cell number and fold expansion are shown in the graph

To generate Figs. 1 and 2, Thy-Tregs were expanded following our previously established protocol [14]. Briefly, isolated cells were seeded at 10^6^ cells/ mL in tissue culture 24 well plate (Corning) of flask (TPP) in X-VIVO 15 (Lonza) supplemented with 5% of human AB serum (BioWest) and activated with TransAct (according to the manufacturing instructions, Miltenyi Biotec) or CTS Dynabeads Treg Xpander (1:1 bead to cell ratio, Thermo Fisher Scientific) in the presence of IL-2 (1000 IU/mL; Proleukin, Novartis), and Rapamycin (100 nM; LC-Laboratories). Cells were re-stimulated at day 12 (at 1:1 bead to cell ratio with CTS Dynabeads Treg Xpander and 1: 100 with TransAct) and expanded up to 24 days. For the process validation runs (PV1 and PV2) and the Engineering run, isolated Thy-Tregs were seeded in the appropriate G-Rex bioreactor (Wilson -Wolf) in X-VIVO 15 (Lonza) supplemented with 5% of human AB serum and activated with CTS Dynabeads Treg Xpander beads at 1:1 bead to cell ratio in the presence of IL-2 (1000 IU/mL; Proleukin, Clinigen), and Rapamycin (100 nM, Miltenyi Biotec). Cells were then restimulated at day 10 (1:1 bead to cell ratio) and day 17 (0.5:1 bead to cell ratio) and cultured up to 23 days.

A full list of reagents used to isolate and expand Thymic -Tregs can be found in Supplementary Table 1.

### Thy-Treg cryopreservation and thawing

For cryopreservation, Thymic-Tregs of the two process validation runs (PV1 and PV2) and the Engineering run were pelleted by centrifugation and resuspended in CryoStor CS10 freezing medium (STEMCELL Technologies) to the desired concentration to formulate the drug product (DP) in a final volume of 10mL. The DP was then transferred into a CryoMACS freezing bag (Miltenyi Biotec). All the remaining cells were frozen in CryoStor CS10 freezing medium and transferred into Cryovials (Thermo Scientific), 1 mL per vial for quality control (QC) testing. Both DP bag and vials were then frozen down in a controlled rate freezer using in-house qualified freezing protocol for freezing T cells. Briefly, the program involved cooling from + 10 °C to -80 °C at a rate of -1 °C per minute. Both the DP bag and cryovials were then transferred to a liquid nitrogen tank. To assess the release criteria purity, potency, viability, and impurities through flow cytometry analysis (Supplementary Table 2) cryopreserved Thy-Tregs were thawed rapidly and diluted in complete medium. The in-house stability assay was performed by thawing the DP bag and the viability was assessed at different time points using the NC200 cell count.

### Flow cytometry analysis

Expanded Thy-Tregs were washed in PBS and stained with LIVE/DEAD^®^ Fixable Near-IR Dead Cell Stain Kit (Thermo Fisher Scientific), according to the manufacturer’s instructions (Thermo Fisher Scientific). Cells were then washed and labelled with a combination of surface molecules as previously described [14]. FOXP3 staining was performed with Foxp3/ Transcription Factor Staining Buffer Set (eBioscience) according to the manufacturer’s instructions. Stained cells were acquired on 5-lasers LSRFortessa X20 (BD Bioscience) and analysed using Flowjo (version 10 for Mac). The phenotype of Thy-Tregs generated during the process validation runs (PV1 and PV2) and the Engineering run was assessed under GMP quality control (QC) testing. Briefly, Thy-Tregs were labelled with LIVE/DEAD^®^ Fixable Near-IR Dead Cell Stain Kit (Thermo Fisher Scientific), according to the manufacturer’s instructions. Cells were then washed and labelled with the following combination of surface molecules: CD3, CD4, CD8, CD25 and CD127. FOXP3 staining was performed with Foxp3/ Transcription Factor Staining Buffer Set (eBioscience) according to the manufacturer’s instructions.

### In vitro suppression assay

**Fig. 3:**
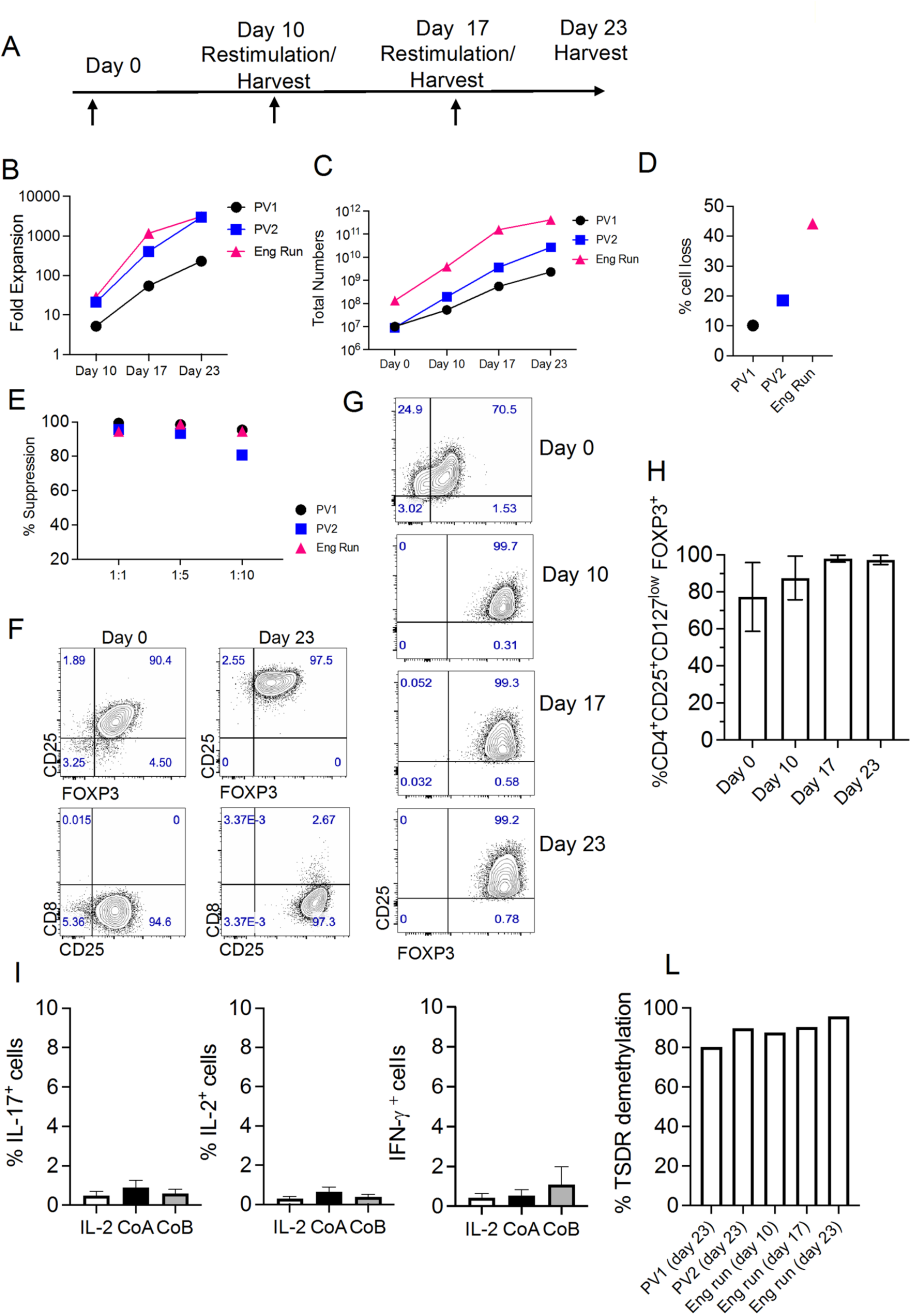
Technology transfer and process validation runs of Thy-Treg expansion protocol. **(A)** Thy-Treg manufacturer protocol. Thy-Tregs are stimulated at day 0 with αCD3/αCD28 CTS Treg Xpander beads at 1:1 cell to bead ratio in the presence of IL-2 and Rapamycin. Cells are cultured in the G-Rex bioreactor between 10 and 23 days. **(B)** Fold expansion and **(C)** total numbers of two laboratory-scale PV runs and a full scale GMP engineering run at each potential harvest time point of the protocol. **D)** Percentage of cell loss observed after bead removal at day 23 in each Thy-Treg preparation **(E)** Suppressive ability of Thy-Tregs labelled with red fluorescent cell linker PKH26 and cocultured with CFSE-labelled effector CD25^−^ T cells at the indicated ratios and stimulated with αCD3/αCD28 beads at 40:1 (cell to bead ratio) for 5 days **(F)** Representative dot plots showing CD25 and FOXP3 expression and CD8 and CD25 co-expression on freshly isolated and expanded Thy-Tregs. **(G)** Representative dot plots and cumulative results **(H)** showing the percentage of CD25 and FOXP3 over the expansion period. Data are expressed as mean ± SEM and are pooled from PV1, PV2 and the Engineering run. **(I)** Cumulative data showing the percentages of IL-17, IL-2 and IFN-γ producing cells after stimulation in the presence of IL-2 only (white bar), Cocktail A (CoA) (IL-2, IL-1β, IL-6 and TGF-β) (black bar) and Cocktail B (CoB) (IL-2, IL-21, IL-23, and TGF- β) (Grey bar). Bars represent the mean ± SEM. Data come from expanded (day 23) Thy-Tregs from PV1, PV2 and the Engineering run. (L) Bar plots showing the percentage of demethylation of the TSDR in PV1, PV2 (day 23) and Engineering run (day 10, 17 and 23)

CD4^+^CD25^−^ T cells (Teffs) were labelled with 2.5 µM CFSE for 4 min at room temperature and activated with αCD3/αCD28 beads (Invitrogen) at 40:1 cell to bead ratio. Teffs were then cultured alone (1 × 10^5^) or cocultured with HLA-A2 mismatched Thy-Treg cells at different ratios in a 96 U-bottom well plate. After 5 days, cells were stained with LIVE/DEAD^®^ Fixable Near-IR Dead Cell Stain Kit (Thermo Fisher Scientific) and an anti-HLA-A2 antibody and acquired on 5-lasers LSRFortessa X20 (BD Bioscience). The percentage of suppression was calculated based on the proliferation of Teffs alone compared with the percentage of proliferation observed in the presence of Thy-Tregs. The suppression assay of Thy-Tregs generated during the process validation runs (PV1 and PV2) and the Engineering run (Fig. 3G) was performed under GMP quality control (QC) testing. Briefly, Thy-Tregs were labelled with red fluorescence cell linker (PKH26) and co-culture with CFSE labelled Teff cells activated with αCD3/αCD28 beads for 5 days.

### TSDR analysis

The percentage of demethylation of the Treg specific demethylated region (TSDR) (CNS2 in FOXP3 gene) was calculated using the PureQuant™ Treg Assay (Thermo Fisher Scientific) according to manufacturer’s instructions. Briefly, genomic DNA was isolated from 10^6^ cells and then subjected to bisulfite conversion followed by qPCR as described by Wieczorek et al. [23].

### Stability assay

Cryopreserved expanded (day 23) Thy-Tregs from the two process validation runs (PV1 and PV2) and Engineering run, were thawed and stimulated with CTS Dynabeads Treg Xpander beads at a bead: cell ratio 1:1 and cultured for 5 days in X-VIVO 5% HS in the presence of cytokine cocktails as previously described [14]. At the end of expansion, expression of IL-2, IFN-γ and IL-17 was assessed after activation of the cells with phorbol-myristste-acetate (PMA, 50 ng/mL, Sigma-Aldrich), Ionomycin (1 µg/mL, Sigma-Aldrich) and BD GolgiStop™ Protein Transport Inhibitor (BD Biosciences) according to the manufacturer’s instruction for 5 h. Cells were then washed in PBS and stained with LIVE/DEAD^®^ Fixable Near-IR Dead Cell Stain Kit (Thermo Fisher Scientific), according to the manufacturer’s instructions (Thermo Fisher Scientific). Cells were washed and labelled with surface molecules CD4 and CD25. Subsequent intracellular staining for FOXP3, IL-2, IFN- γ and IL-17 was performed with Foxp3/Transcription Factor Staining Buffer Set (eBioscience) according to the manufacturer’s instructions. Stained cells were acquired on 5-lasers LSRFortessa X20 (BD Bioscience) and analysed using Flowjo (version 10 for Mac).

### Statistical analysis

Statistical tests used are indicated in the figure legends and were performed using GraphPad Prism 10.

## Results

### Clinical -grade thymus tissue digestion to isolate and expand Thy-Tregs

To translate the methodology of thymus tissue digestion into a GMP-compliant process, we tested an alternative method using solely the GentleMACS dissociator to standardise the homogenisation of the tissue without performing any enzymatic digestion, previously used in our protocol [14]. In fact, the use of enzymes may complicate the GMP process development depending on the source of the enzyme and batch to batch variations associated with both bioproducts. After performing an initial manual dissociation of the thymus tissue with scissors, we compared each of the two methods in parallel, namely the enzymatic digestion followed by the GentleMACS dissociator and the use of the GentleMACS dissociator only by analysing the yield of total thymocytes obtained at the end of the two processes. As shown in Supplementary Fig. 1A, no significant differences in the thymocyte yield were observed suggesting that the mechanic dissociation per se represents a feasible GMP approach. Here we need to mention that only few samples were processed to conduct such analysis. However, the comparison of the mean of thymocytes yield previously obtained (mean = 0.625 × 10^9^/gr) [14] with the thymocyte yield (mean = 1.0 × 10^9^/gr, Table 1) observed by processing a comparable number of thymuses without performing the enzymatic digestion step, supports the use of GentleMACS dissociator only.

In addition, we found a mean yield of 4.54 × 10^6^ Tregs/gr which was higher than what we obtained with the original enzymatic digestion process (mean = 2.02 × 10^6^ Tregs/gr [14])

To complete our comparison, we performed a full expansion process with the protocol previously described [14] starting with the two different preparations of thymocytes. Briefly, thymocyte suspensions were processed through the two-step magnetic bead separation protocol, namely depletion of CD8^+^ cells followed by CD25^+^ enrichment. As shown in Supplementary Fig. 1B and 1 C the isolation process was comparable between the two samples as well as the percentage of CD25^+^FOXP3^+^ Thy-Tregs obtained at the end of the purification (> 75%) and the expansion process (> 90%). Accordingly, both Thy-Treg preparations retained the expression of surface homing receptors such as CD62L and CXCR4 [14, 20] and they were also all positive for key Treg markers such as Helios, CTLA-4 and TIGIT (Supplementary Fig. 1D). The two populations of Thy-Tregs showed comparable expansion rate which was greater than 75 (Supplementary Fig. 1E) following 24 days of expansion alongside their suppressive ability (Supplementary Fig. 1F). Altogether these data confirm the quality of our final cell product upon the exclusion of the enzymatic digestion.

**Table 1:** Characteristics of thymus tissues processed with GentleMACS dissociator in the absence of enzymatic digestion

Donor	Weight (gr)	Age (months)	Thymocytes/gr (x10^9^)	Processed Thymocytes (x10^6^)	Post CD8 depletion (x10^6^)	Post CD25 enrichment (x10^6^)	Tregs/gr (x10^6^)
Donor 1	2	9	0.5	1000	60	2.95	1.475
Donor 2	3.93	1	1.8	1000	67.2	2.28	4.104
Donor 3	1.62	13	1.13	1500	100	3	2.26
Donor 4	2	60	0.48	960	115	4	2
Donor 5	3.1	36	0.8	1500	223	10	5.3
Donor 6	3.39	5	1.1	1500	150	9	6.6
Donor 7	2.74	48	0.71	1500	100	8.55	4.047
Donor 8	4	0.3	0.375	1600	150	15	3.5
Donor 9*	18.49	2	1.081	20000	821	133	7.18
Donor 10	4	0.3	1.75	1600	100	7	7.65
Donor 11	4	4	1.4	1200	80	5	5.83
**Mean**	**4.48**	**16.23**	**1.0**	**3032.7**	**178.74**	**18.16**	**4.54**
Range	1.62–18.49	0.3–60	0.375–1.8	960–20000	60–821	2.28–133	1.47–7.6

* Engineering run

### CTS Dynabeads but not TransAct efficiently expand Thy-Tregs for clinical application

Our previous established protocol [14] has shown the capability of expanding large numbers of Thy-Tregs for clinical applications by using GMP-grade αCD3/αCD28 coated beads from Miltenyi Biotec (ExpAct Treg kit) already used by us and others in several clinical trials [8, 24, 25]. As ExpAct beads have been recently discontinued, this has raised the need of alternative activation approaches to expand Thy-Tregs for the manufacturing process. We therefore compared the expansion rate of Thy-Tregs stimulated with either CTS Dynabeads Treg Expander, which similarly to the ExpAct beads are paramagnetic hence their removal is required before the final formulation or T cell TransAct, a CD3/CD28 polymeric nanomatrix designed to expand T cell populations which can be easily removed by simple washing. We used the CTS Dynabeads according to a validated bead: cell ratio (1:1) previously tested by us [26, 27] whereas the TransAct have been added to the cell culture at the concentration indicated in the manufacture’s instruction.

We found that the fold expansion with TransAct was almost indiscernible compared to what we obtained with CTS Dynabeads Treg Xpander beads either during the first 12 days of expansion or after restimulation (Fig. 1A). Accordingly, Thy-Tregs activated with TransAct were less suppressive compared to the CTS expanded Tregs (Fig. 1B) and they exhibited as well lower CD25 and FOXP3 co-expression at the end of the expansion (Fig. 1C and D). Finally, those Thy-Tregs activated with TransAct maintained high expression of some key Treg markers such as, Helios and CTLA-4 but at the end of the expansion period they started to downregulate the expression of surface markers such as TIGIT and in a significant manner HLA-DR, CD62L, and CXCR4 whose expression remained high in the CTS-expanded Thy-Tregs (Fig. 1E and F).

Therefore, based on the reasons outlined above and the comparable fold expansion and phenotype of Thy-Tregs expanded with CTS Dynabeads to those previously achieved using ExpAct beads (Supplementary Fig. 1E), we selected the CTS Dynabeads Treg Xpander beads for validation of our Thy-Treg expansion protocol for manufacturing.

### Small-scale validation of Thy-Treg expansion protocol

Having established a GMP-compliant dissociation process alongside the use of CTS Treg Xpander beads, we then confirmed the reproducibility of the new methodology by processing additional thymus samples. Flow cytometry analysis revealed high purity of the final product in terms of CD25 and FOXP3 expression (mean 90%) compared to Thy-Tregs isolated at day 0 (mean 73.2%) (Fig. 2A and B). Accordingly, both expansion rate and suppressive ability (Fig. 2C and D) were consistent to what was previously observed (Supplementary Fig. 1E and F) confirming therefore the validation of the new dissociation and activation approach.

A critical point to manufacturing large number of stable Tregs while preserving their suppressive function and stability is the choice of the restimulation time. In previous publications, Tregs purified from blood or leukapheresis have been restimulated between day 7 and 8 [24, 28, 29] and multiple restimulations have been included to reach the final formulation according to the patients to treat, cryopreservation and time of infusion. Recently, it has been shown that thymic Tregs restimulated at day 9, after returning to their resting state, have a higher growth compared to those restimulated at day 7 and that expanded Tregs cryopreserved 1–3 days following restimulation maintained phenotypic and functional properties [20]. It should be mentioned here that as the cell growth depends on the culture systems such as flasks, expansion bags or G-Rex bioreactors, the frequency of feedings and accordingly the timing for the cells to reach their resting state dramatically differ.

We transferred the expansion protocol to the G-Rex bioreactor to scale-up Thy-Treg manufacturing process and we compared the effect of two different restimulation time points, namely day 7 as published by others and day 10, which is between the restimulation time (day 9) suggested by Levings group [20] and day 12 previously published by us [14] (Fig. 2E). As shown in Fig. 2F, Thy-Tregs cultured in a G-Rex bioreactor and restimulated at day 10 showed a fold expansion nearly 2.4 times higher compared to those Thy-Tregs restimulated at day 7, supporting therefore the concept that returning to resting size prior restimulation favours a superior Treg expansion.

### Scale-up validation of Thy-Treg expansion protocol for manufacturing

Based on this evidence and on the rapid expansion of Thy-Tregs in the G-Rex bioreactor, we modified our previous protocol into the current clinical protocol shown in Fig. 3A. As before [14], Thy-Treg culture was supplemented with IL-2 and Rapamycin over the time. However, compared to our previous expansion protocol, the expansion rate obtained in the G-Rex bioreactor offered the possibility to reach high cell numbers by day 10 (Fig. 3C). Considering the highly variable expansion rate among donors and the unpredictable amount of starting tissue material, we have introduced two additional restimulations at day 10 and 17 at cell/bead ratio 1:1 or 1:0.5 respectively to culture the cells up to 23 days.

As part of the technology transfer to the manufacturing process, we performed two laboratory-scale process validation runs (PV1 and PV2) and a full scale GMP engineering run (Eng run) to demonstrate the reproducibility of our expansion protocol. As shown in Fig. 3B our Thy-Treg preparations showed a fold expansion greater than 200 after 23 days of expansion (range 230–3127) and accordingly large cell numbers were obtained (Fig. 3C). As previously mentioned, using paramagnetic beads implies their removal before the final formulation and cryopreservation. As shown in Supplementary Table 2 we achieved a successful manual DynaMag bead removal across the three Thy-Treg cell preparations though the percentage of cell loss was highly variable (Fig. 3D). We then tested the suppressive ability and the phenotype of the final cell product after thawing. All the Thy-Treg preparations were highly suppressive (Fig. 3E), and they contained only a small percentage of CD4^+^CD8^+^DP Tregs which, as shown by Correa-Rocha et al. [15], were also highly positive for CD25 expression (Fig. 3F) [15]. Expanded Thy-Tregs were showing, compared to freshy-isolated Thy-Tregs (Supplementary Fig. 2), more than 90% co-expression for CD25 and FOXP3 (Fig. 3G and H) at each potential harvest time point suggesting therefore the quality of our expanded Thy-Treg cell products. More importantly, Thy-Tregs expanded for 23 days and cultured, upon cryopreservation and thawing, under Th-17 skewing conditions [26, 30] maintained high expression of FOXP3 (Supplementary Fig. 3) alongside their inability to produce IL-2, IL-17 and IFN-γ (Fig. 3I). To further confirm the stability of our cell product at each potential harvest time point of our expansion protocol (Fig. 3A) we tested the demethylation status of FOXP3 CNS2, a Treg-specific demethylated region (TSDR). As shown in Fig. 3L the level of demethylation in our Thy-Treg preparations from PV1, PV2 and the Engineering run following expansion, cryopreservation and thawing was over 80% confirming therefore their epigenetic stability.

### Release criteria for expanded Thy-Tregs

As per United Kingdom regulatory body Medicines and Healthcare products Regulatory Agency (MHRA) requirements, prior to release the final cell product for patient administration an extensive quality and safety testing was conducted on expanded Thy-Tregs harvested at three different time points (Supplementary Table 2). It was found that the final formulation performed at each harvest point met and exceed all the MHRA approved release criteria. As another relevant quality control measurement is the stability of the final drug product from the point of cryo-storage, we assessed both the phenotype and the functionality of our cell product soon after manufacture (Baseline) and at 12 months post cryopreservation. We showed evidence that expanded Thy-Tregs preserved their immunophenotype and suppressive ability in accordance with the MHRA specifications (Supplementary Table 3). In addition, we checked the viability of the final drug product over several hours after thawing, and we found that our cells maintained high viability over the time which started to slightly decrease under the specification (70% of viability) 6 h post thawing (Supplementary Fig. 4). All together these data confirm the efficacy of our cryopreservation approach which guarantees a stable and functional Treg cell product.

## Discussion

Here we demonstrated the feasibility of our manufacturing GMP protocol to efficiently expand high number of Tregs isolated from paediatric thymuses which has been now approved by the MHRA and will be administered for the first time in UK to children (range 0.5–15 years) undergoing heart transplant (ATT-Heart, ISRCTN15374803). Compared to the current manufacturing protocol used in the clinical trial THYTECH (NCT04924491), that aimed to investigate safety of infusing Thy-Tregs into infants under 2 years old following heart transplant [17], here we have demonstrated the capability to manufacture a thymic Treg cell product for a broad age range of children. Our GMP-expansion protocol also represents a feasible approach to develop an “off-the-shelf” cell therapy product for future clinical applications by cryopreserving expanded cells.

Through several implementations, we reached a successful GMP protocol designed to overcome the limitation of the size of the thymus tissue, which could likely be the case in children older than 2 years of age. In fact, thymus tissue grows after birth, reaches maximal size after the first few years and involutes by puberty [31, 32]. This implies that the starting number of CD25^+^ cells to seed in the appropriate G-Rex bioreactor could dramatically vary according to the thymus size and a longer expansion will be most likely required to reach the drug product for the eldest population.

Considering that thymus tissue is bigger in size in infants and a mean yield of 4.54 × 10^6^ Thy-Tregs, as shown here, can be obtained per gr of tissue, there is no need to obtain large number of expanded Thy-Tregs to treat children under 2 years. It follows that a short cell expansion is sufficient to guarantee a final product of Thy-Treg expressing high levels of FOXP3 and CD25. This has therefore warranted the use of TransAct as activation reagents in the THYTECH trial [15] avoiding therefore cell loss associated with the magnetic bead removal. Conversely, this strategy has proven to be ineffective for our manufacturing process in terms of numbers of cells reached at the end of expansion. In fact, as shown by us and others [15, 20] the fold expansion obtained with TransAct was not comparable to the other activation methods though the cells activated for a short time maintained the expression of key Treg cell markers. However, our results show that over the time, the expression of homing and activation markers such as CD62L and CXCR4 and the Treg stability marker TIGIT started to be downregulated suggesting that cells re-stimulated with TransAct might potentially lose their stability during the culture. The decision to use CTS beads was also supported by comparable results observed with ExpAct beads (Supplementary Fig. 1), which had been successfully used to expand Tregs cells for clinical application prior to their discontinuation [8, 24].

In sharp contrast with THYTECH trial, we show here that using the CTS Dynabeads Treg Xpander allowed us to manufacturing high doses of Thy-Tregs by 10 days with the possibility to restimulate the cells and continue the cell culture up to 23 days in the event of limited starting tissue material. The results of the full-scale engineering run (Supplementary Table 2), in particularly the quality and the potency of our Thy-Tregs harvested at Day 10, 17 and 23 confirm the robustness of our expansion protocol and its consistency across different harvest time points. Our data support the feasibility of formulating a drug product that consistently maintains high levels of FOXP3 and CD25 expression together with strong suppressive function regardless of harvest timing ensuring the possibility to treat a broad age range of children.

We acknowledge that a limitation of this approach is the cell loss during the bead removal required before the final formulation. The percentage of cell loss observed was highly variable, although limited so far to three runs. We need here to point out that by obtaining large numbers of Thy-Tregs at each time point of the manufacturing process (day 10 mean = 1.38 × 10^9^, day 17 mean = 5.34 × 10^10^ and day 23 mean = 1.4 × 10^11^) the cell loss after bead removal will not affect the success of the patient dose formulation as well as the availability of samples for quality control (QC) testing. The striking feature of the proposed manufacturing protocol is the robust method developed to cryopreserve expanded Thy-Tregs in a controlled rate freezer which to our knowledge, has not been previously described. In fact, the impact of the cryopreservation has always been the niggle of Treg cell therapy due to its potential detrimental effect on Treg phenotype, viability, and functionality [21, 33, 34].

**Fig. 4:**
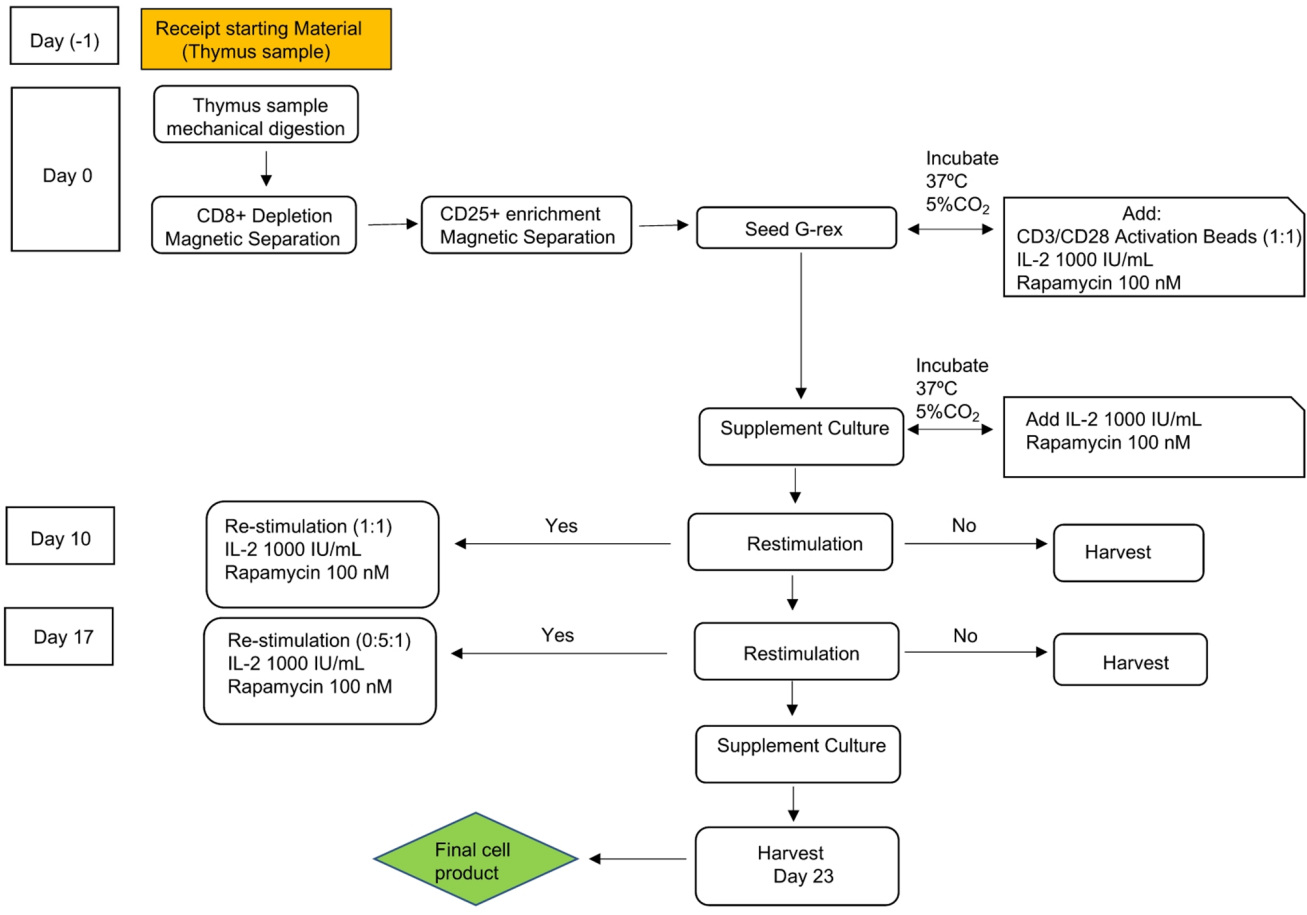
Schematic overview of the manufacturing process

The QC testing performed on the drug product after thawing demonstrated that our cryopreserved cells were more than 94% viable, and they retained a stable phenotype alongside their suppressive ability. Such stable immunophenotype and functionality was still observed 12 months after cryopreservation which, from a practical perspective, will give more flexibility in terms of planning the therapy and timing of infusion. The stability of our Thy-Treg product was further confirmed under inflammatory conditions where Thy-Tregs cryopreserved and thawed after 12 months, did not produce IL-2, IFN-γ or IL-17 and more importantly maintained a demethylated TSDR at each potential harvest time point. Overall, here we provide an optimised strategy to cryopreserve expanded Thy-Tregs in conjunction with a GMP- Thy-Treg expansion process (Fig. 4).

To conclude, the improved quality and amount of Thy-Tregs obtained with our manufacturing protocol will allow to infuse a broad age range of children including those requiring interim mechanical support implantation procedure, who may have deferred transplant surgery, with the possibility to provide more than one dose to potentially achieve indefinite immunological tolerance after the transplant and to use expanded Thy-Tregs for future clinical applications.

## Electronic supplementary material

Below is the link to the electronic supplementary material.

Supplementary Material 1

## Data Availability

The data that support the findings of this study are available from the corresponding author, [G.F.], upon reasonable request.

## Abbreviations

DP: Drug product
Eng Run: Engineering run
GMP: Good Manufacture Practise
GOSH: Great Ormond Street Hospital
MHRA: Medicines and Healthcare products Regulatory Agency
PBS: Phosphate Buffer Saline
PV run: Process Validation run
QC: Quality Control
Regulatory T cells: Tregs
Thy: Tregs-Thymic Tregs
