# Supplementary material for: Clinical grade expansion protocol for the manufacture of thymus-derived Treg cells for clinical application
Source: J Transl Med. 2025 Jun 3;23:620. doi: 10.1186/s12967-025-06561-9 (PMC12131477; doi:10.1186/s12967-025-06561-9)
Supplement: Supplementary file 1 — Supplementary Material 1 [file 12967_2025_6561_MOESM1_ESM.docx]

**Title**

**Clinical grade expansion protocol for the manufacture of Thymus-derived Treg cells for clinical application**

**List of authors**

Giorgia Fanelli^1^, Philippa Marks^4^*, Apoorva Aiyengar ^2,3^, Marco Romano^1^, Sakina Gooljar^4^ Sandeep Kumar ^4^, Michael Burch^2,3^ and Giovanna Lombardi ^1^

^1^Peter Gorer Department of Immunobiology, School of Immunology and Microbial Sciences, King’s College, London, United Kingdom

^2^ Department of Cardiology, Great Ormond Street Hospital NHS Foundation Trust, London, United Kingdom

^3^ Research Department of Children’s Cardiovascular Disease, Institute of Cardiovascular Science, University College London, London, United Kingdom

^4^ Advanced Therapy Manufacturing (GMP) Unit Guy’s and St Thomas’ NHS Foundation Trust and King’s College London Clinical Research Facility, London, United Kingdom

**Supplementary Figure 1.**

**Fig. S1.** **Representative comparison of thymus digestion approaches through a full isolation and expansion process. (A)** Comparison of yield of thymocytes obtained with enzymatic digestion followed by the GentleMACS dissociator (black bar) or the use of the GentleMACS dissociator only (white bar). Bars represent the mean ± SEM. Data are pooled from 3 independent experiments (n = 3 different donors) **(B)** Representative two-step magnetic bead selection process showing the total number of CD8^-^ and CD25^+^ cells obtained through the enzymatic digestion followed by the GentleMACS dissociator (black bar) or the use of the GentleMACS dissociator only (white bar). **(C)** Representative dot plots showing the gating strategy used to gate CD25^+^FOXP3^+^ cells obtained with the two thymus digestion approaches at the end of the isolation step and cell culture. **(D)** Representative histograms showing the expression of the indicated markers on Thy-Tregs isolated following the two different digestion and expanded for 24 days **(E)** Comparison of fold expansion of isolated thymic Tregs activated with αCD3/αCD28 MACS GMP ExpAct beads at 1:1 cell to bead ratio) in the presence of IL-2 and Rapamycin. Cells were restimulated at day 12 and cultured for 24 days. **(F)** Suppression assay of Thy-Tregs cocultured with CFSE-labelled effector CD25^-^ T cells, HLA-A2 mismatched to Tregs (left panel), at the indicated ratios and stimulated with a αCD3/αCD28 beads at 40:1 (cell/bead) ratio for 5 days. Suppression of CD25^-^ T proliferation was determined by division index**.**

**Supplementary Figure 2**

**Fig. S2.** **Viability, phenotype and suppressive ability of fresh Thy-Tregs from PV1 and PV2 runs. (A)** Representative dot plots showing the gating strategy used to gate CD25^+^FOXP3^+^ cells obtained at day 23. Percentage of viability **(B)** and CD25 and FOXP3 expression **(C)** observed at day 23 in PV1 and PV2 Thy-Treg preparations. **(D)** Suppression assay of PV1 and PV2 Thy-Tregs cocultured with CFSE-labelled effector CD25^-^ T cells, HLA-A2 mismatched to Tregs (left panel), at the indicated ratios and stimulated with a αCD3/αCD28 beads at 40:1 (cell/bead) ratio for 5 days. Suppression of CD25^-^ T proliferation was determined by division index**.**

**Supplementary Figure 3.**

**Fig. S3. Thy-Treg stability in the presence of pro-inflammatory cytokines.** Representative dot plots showing the percentage of FOXP3 expression and IFN-γ, IL-2 and IL-17 producing cells following freezing and thawing. Expanded Thy-Tregs (day 23) and CD4^+^CD25^-^ T cells (Teff, positive control) have been activated in the presence of IL-2 only, Cocktail A (CoA) (IL-2, IL-1β, IL-6 and TGF-β) and Cocktail B (CoB) (IL-2, IL-21, IL-23, and TGF- β) for 5 days. All the cell preparation have been then stimulate with PMA (50 ng/mL) Ionomycin (1µg/mL) and BD GolgiStop™ Protein Transport Inhibitor (BD Biosciences) according to the manufacturer’s instruction for 5 hours.

**Supplementary Figure 4.**

**Fig. S4. In-use stability assay.** The drug product was cryopreserved in Cryostor CS10 freezing medium and thawed 5 weeks post cryopreservation. Time-point viability (%) was assessed using NC200 cell count. Spec= specification; DP= Drug product

**Supplementary Table 1.**

|  | RUO | GMP | Source |
| --- | --- | --- | --- |
| Tissue Dissociation |  |  |  |
| Dissociator | GentleMACS Dissociator | GentleMACS Octo Dissociator | Miltenyi Biotec |
| Magnetic cell separator | QuadroMACS Separator and LS columns | CliniMACS plus System and tubing Set | Miltenyi Biotec |
| CD8 Beads  CD25 Beads | CD8 Microbeads II  CD25 Microbeads II | CliniMACS CD8 Reagent  CliniMACS CD25 Reagent | Miltenyi Biotec |
| Washing Buffer | MACS Buffer  PBS/2 mM EDTA/0.5% BSA | CliniMACS Buffer PBS/EDTA/0.5% HSA | Miltenyi Biotec |
| Culture medium | X-VIVO 15 | GMP X-VIVO 15 | Lonza |
| IL-2 | Proleukin | Proleukin | Novartis  Clinigen |
| Freezing medium | 10% DMSO  90% HS | Cryostor CS10 | STEMCELL Technologies |
| anti-CD3/anti-CD28 Beads | Dynabeads  TransAct  ExpAct | CTS Treg Xpander | ThermoFisher  Miltenyi Biotec |
| Culture Vessels | 24 well plate  25/75 cm^2^ flask  G-Rex 10/100 | G-Rex 10  Grex 100 | Corning  TPP  Wilson Wolf |

RUO= Research Use Only; GMP= Good Manufacturing Practice

**Table S1.** List of reagents and equipment used during the process development and manufacturing process.

**Supplementary Table 2.**

**Table S2.** Realise criteria of the final Thy-Treg product and results of the full scale GMP-engineering run

**Supplementary Table 3.**

**Table S3**. Phenotype and Suppression assay result of Thy-Tregs tested 12 months after cryopreservation
